# The Role of Scientific Collections in Scientific Preparedness

**DOI:** 10.3201/eid2108.150423

**Published:** 2015-08

**Authors:** Diane DiEuliis

**Affiliations:** US Department of Health and Human Services, Office of the Assistant Secretary for Preparedness and Response, Washington, DC, USA

**Keywords:** Diseases, scientific collections, scientific samples, global health, health security, science preparedness, workshop

## Abstract

Building on the findings and recommendations of the Interagency Working Group on Scientific Collections, Scientific Collections International (SciColl) aims to improve the rapid access to science collections across disciplines within the federal government and globally, between government agencies and private research institutions. SciColl offered a novel opportunity for the US Department of Health and Human Services, Office of the Assistant Secretary for Preparedness and Response, to explore the value of scientific research collections under the science preparedness initiative and integrate it as a research resource at each stage in the emergence of the infectious diseases cycle. Under the leadership of SciColl’s executive secretariat at the Smithsonian Institution, and with multiple federal and international partners, a workshop during October 2014 fully explored the intersections of the infectious disease cycle and the role scientific collections could play as an evidentiary scientific resource to mitigate risks associated with emerging infectious diseases.

During the past decade, public health emergencies have challenged the preparedness and response of government agencies, hospitals and clinics, public health professionals, and academic researchers in the United States and abroad. Several devastating infectious diseases have been transmitted to human populations from different animal species, including severe acute respiratory syndrome, pandemic influenza A(H1N1), and, most recently, Ebola virus disease in West Africa. According to the World Health Organization and the United States Centers for Disease Control and Prevention, as many as 60% of emerging infectious diseases originated in animals (*1*), and another 17% originated from insects or other types of vectors (*2**,**3*).

When such outbreaks occur, epidemiologists, public health workers, researchers, and clinicians begin research that is tied to the infectious disease outbreak cycle (Figure). When they isolate and identify the infectious agent, they perform genetic analyses, use diagnostic methods, develop and use potential medical countermeasures, and guide practices for best medical treatment. Even though the infectious disease outbreak lifecycle (Figure) might seem routine, each outbreak is different and presents unique research challenges to the mitigation of the spread of disease and to the protection of human lives.

**Figure:**
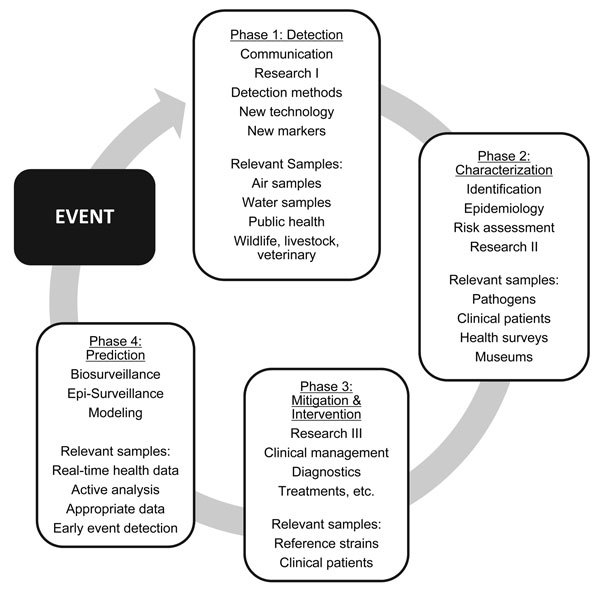
Cycle of tasks for public health investigation of infectious disease outbreaks.

With this understanding, the US Department of Health and Human Services (HHS) Office of the Assistant Secretary for Preparedness and Response (ASPR) has undertaken a science preparedness initiative. ASPR’s experiences have emphasized the importance of the ability to perform rapid scientific research during a limited timeframe when responding to emerging infectious disease outbreaks or other public health emergencies (*4*). This office has to be prepared to answer timely questions during a response, and research results can enable a more educated and informed response to similar future events, maximizing recovery. The science preparedness effort is designed to ensure that such research needs are prioritized and to support needed infrastructure for such research.

Knowing what tools and resources are available at any given point during a response and making them available to researchers is one of the goals of science preparedness. Scientific collections span a wide range of scientific study and disciplines and include various objects from lunar rocks to bacteria. Collections are widely distributed across federal agencies and throughout the country at state and local levels. These collections form a base of support for scientific study that informs regulatory, management, and policy decisions. In fact, the Office of Science and Technology Policy in the department of the Executive Office of the President created the Interagency Working Group on Scientific Collections to facilitate policy development and identify a systematic approach to safeguarding these valuable scientific resources, making them more readily available and accessible to the research community (*5*).

Building on the findings and recommendations of the Interagency Working Group on Scientific Collections, Scientific Collections International (SciColl; http://www.scicoll.org) aims to improve the rapid access to science collections across disciplines, not just within the federal government but also globally, between government ministries and private research institutions. Through a joint partnership, the Interagency Working Group on Scientific Collections, SciColl offered a novel opportunity for ASPR to explore the value of scientific research collections under the science preparedness initiative and integrate it as a research resource at each stage in the emergence of infectious diseases cycle.

Under the leadership of the SciColl executive secretariat at the Smithsonian Institution, and with multiple federal and international partners, attendees of a workshop held on October 23 and 24, 2014, fully explored the intersections of the infectious disease cycle and the role scientific collections could play as an evidentiary scientific resource to mitigate the risks associated with emerging infectious diseases. During this dynamic and collaborative forum, the case studies presented exemplified how specific collections of mammals, parasites, and other vectors and reservoirs of pathogens provide evidence and understanding of disease emergence in human populations. Participants articulated needs and possible methods to capitalize on the use of collections, including their practical research applications and also policy issues surrounding their use, during outbreaks. As highlighted in the published workshop report (6), specific recommendations for data management, innovative approaches to cross-disciplinary research, and communication and sample sharing were identified. The achievement of these critical milestones will maximize the use of scientific collections to their greatest benefit.

The findings from the workshop highlight the importance of management practices, access, and use of scientific collections in detecting, characterizing, mitigating, and predicting emerging infectious diseases. These findings were identified in the midst of one of the most severe Ebola outbreaks in history, and applying their utility will benefit future efforts to respond to public health emergencies and save lives. Employees of ASPR will continue to work toward the greater collaboration and integration of scientific collections in mitigating, preventing, responding to, and preparing for emerging infectious diseases. By emphasizing the importance of science-supported research and strengthening initiatives like science preparedness, we can develop policies and systems that fully realize the practical application of scientific collections.

We encourage private and public research institutions to get involved with SciColl and engage in the global effort to work toward a more systematic approach for sharing, managing, and using scientific collections. Similar to that of the work of the recently launched Network Integrated Biocollections Alliance, many initiatives all over the world are being created where scientists and researchers have articulated a need for greater collaboration in digitally capturing and sharing scientific specimens (http://www.niballiance.org/). For this growing movement to be successful, it needs to be one that incorporates a whole of community strategy, where every institution, agency, and scientific discipline is vested in the improvement and development of this vast network. The staff of ASPR look forward to our continued collaboration with SciColl and other partners in the future to strengthen engagement with the stewards of scientific collections at home and abroad.
